# Predictors of humoral response to SARS-CoV-2 mRNA vaccine BNT162b2 in patients receiving maintenance dialysis

**DOI:** 10.1017/ash.2022.31

**Published:** 2022-03-23

**Authors:** Tingting Li, Sumanth Gandra, Kimberly A. Reske, Margaret A. Olsen, Silvana Bommarito, Candace Miller, Karl G. Hock, Claire A. Ballman, Christina Su, Na Le Dang, Jennie H. Kwon, David K. Warren, Victoria J. Fraser, Christopher W. Farnsworth, for the Centers for Disease Control and Prevention Epicenters Program

**Affiliations:** 1 Division of Nephrology, Department of Medicine, Washington University School of Medicine, Saint Louis, Missouri; 2 Division of Infectious Diseases, Department of Medicine, Washington University School of Medicine, St. Louis, Missouri; 3 Department of Pathology and Immunology, Washington University School of Medicine, St. Louis, Missouri; 4 Department of Pediatrics, Washington University School of Medicine, St. Louis, Missouri

## Abstract

**Objective:**

Patients on dialysis are at high risk for severe COVID-19 and associated morbidity and mortality. We examined the humoral response to SARS-CoV-2 mRNA vaccine BNT162b2 in a maintenance dialysis population.

**Design:**

Single-center cohort study.

**Setting and participants:**

Adult maintenance dialysis patients at 3 outpatient dialysis units of a large academic center.

**Methods:**

Participants were vaccinated with 2 doses of BNT162b2, 3 weeks apart. We assessed anti–SARS-CoV-2 spike antibodies (anti-S) ∼4–7 weeks after the second dose and evaluated risk factors associated with insufficient response. Definitions of antibody response are as follows: nonresponse (anti-S level, <50 AU/mL), low response (anti-S level, 50–839 AU/mL), and sufficient response (anti-S level, ≥840 AU/mL).

**Results:**

Among the 173 participants who received 2 vaccine doses, the median age was 60 years (range, 28–88), 53.2% were men, 85% were of Black race, 86% were on in-center hemodialysis and 14% were on peritoneal dialysis. Also, 7 participants (4%) had no response, 27 (15.6%) had a low response, and 139 (80.3%) had a sufficient antibody response. In multivariable analysis, factors significantly associated with insufficient antibody response included end-stage renal disease comorbidity index score ≥5 and absence of prior hepatitis B vaccination response.

**Conclusions:**

Although most of our study participants seroconverted after 2 doses of BNT162b2, 20% of our cohort did not achieve sufficient humoral response. Our findings demonstrate the urgent need for a more effective vaccine strategy in this high-risk patient population and highlight the importance of ongoing preventative measures until protective immunity is achieved.

It is well recognized that patients on maintenance dialysis are at a significantly higher risk for novel coronavirus disease 2019 (COVID-19) and associated morbidity and mortality.^1,2^ Vaccination against severe acute respiratory syndrome coronavirus 2 (SARS-CoV-2) to prevent COVID-19 or ameliorate the severity of infection has been strongly advocated for patients on dialysis. Although mRNA vaccines against SARS-CoV-2 have been shown to be highly effective at preventing COVID-19 and related serious outcomes, including hospitalizations and death,^3–5^ patients on dialysis were generally excluded from, or underrepresented in clinical trials, and whether SARS-CoV-2 vaccination offers the same degree of protection in the dialysis population remains unclear.

Multiple studies have examined the immune response to SARS-CoV-2 mRNA vaccines in patients receiving dialysis, demonstrating relatively high seroconversion rates and varying degrees of humoral and cellular immune response after 2 doses of SARS-CoV-2 mRNA vaccine.^6–12^ Most of these studies were performed in Europe and Israel and consisted largely of white patients. Patients of Black race make up >30% of the United States dialysis population,^13^ and they have been disproportionally affected by the COVID-19 pandemic, with an increased risk for COVID-19 and related deaths compared to their White counterparts.^14,15^ As immunogenicity to vaccines may vary according to race and ethnicity,^16^ better understanding of vaccine response in Black patients on dialysis may help inform future SARS-CoV-2 vaccine strategies in this highly vulnerable population. In this study, we examined the humoral response to 2 doses of SARS-CoV-2 mRNA vaccine BNT162b2 in a predominantly Black dialysis population at a large Midwest academic institution, and we assessed risk factors associated with insufficient vaccine response.

## Materials and methods

This study was conducted at 3 outpatient dialysis units at Washington University School of Medicine (WUSM) in St. Louis, Missouri, and it was approved by the WUSM Human Research Protection Office. In February 2021, a SARS-CoV-2 vaccination program was implemented at these dialysis units, and 2 doses of the SARS-CoV-2 mRNA vaccine BNT162b2 were offered to all patients, given 3 weeks apart. Adult patients on maintenance hemodialysis (HD) or peritoneal dialysis (PD) who received ≥1 dose of the vaccine between February and April of 2021 and provided written informed consent were included in the study.

Demographic, clinical, and laboratory data at baseline (prior to first dose of the vaccine) were extracted from the electronic medical records and data were stored in a REDCap database. History of COVID-19 was defined as documentation of a positive PCR for SARS-CoV-2 at any time prior to the first dose of SARS-CoV-2 vaccination. Comorbidities were considered independently, and a previously validated end-stage renal disease (ESRD) comorbidity index score was used as an estimate of underlying disease burden.^17^ This score was calculated by assigning a numerical weight to each of the following comorbid conditions: atherosclerotic heart disease, congestive heart failure, cerebrovascular accident/transient ischemic attack, peripheral vascular disease, other cardiac disease, chronic obstructive pulmonary disease, gastrointestinal bleeding, liver disease, dysrhythmia, cancer, and diabetes.

A blood sample was obtained ∼4–7 weeks after the second vaccine dose, and immunoglobulin G (IgG) antibodies against the spike protein of SARS-CoV-2 (anti-S) were measured using the AdviseDx SARS-CoV-2 IgG II assay (Abbott, Abbott Park, IL). According to the manufacturer, a result is positive if anti-S is ≥50 AU/mL; patients with an anti-S below this level were considered nonresponders in our study. In the absence of a known antibody threshold that correlates with a protective humoral response, an anti-S threshold of 840 AU/mL was chosen as the cutoff for sufficient response in this study, and patients with an anti-S between 50 AU/mL and 839 AU/mL were considered low responders. The anti-S level of 840 AU/mL from this assay correlates with a neutralizing antibody titer of 1:250, a titer that was considered acceptable by the Food and Drug Administration for use in the manufacture of high-titer COVID-19 convalescent plasma.^18^ A neutralizing antibody titer of 1:250 has also been previously used to define a sufficient serological response.^19^ Finally, the anti-S level of <840 AU/mL is close to the lowest quartile observed in our study (<1,104 AU/mL).

Descriptive and univariate data analyses were performed using the χ^2^ test, the Fisher exact test (for variables with cell size <5), univariate Poisson regression, and Mann-Whitney *U* test, as appropriate. A multivariable generalized linear model with a log link and robust standard errors was used to determine independent risk factors for insufficient vaccine response, with calculation of relative risks.^20^ *P* < .05 was considered statistically significant. We used SPSS version 27 software (IBM, Armonk, NY) and SAS version 9.4 software (SAS Institute, Cary, NC) for statistical analyses.

## Results

Informed consent was obtained, and blood specimens were collected from 181 patients on maintenance dialysis, of whom 157 (87%) received in-center HD and 24 (13%) were on PD. Eight patients (4.4%) had received only 1 dose of BNT162b2 as of the blood specimen collection date. The median anti-S level was significantly higher in patients who received 2 doses compared to 1 dose of the vaccine (4,100 AU/mL vs 191 AU/mL; *P* = .02). Thus, those who only received a single vaccine dose were excluded from further analyses.

**Fig. 1.**
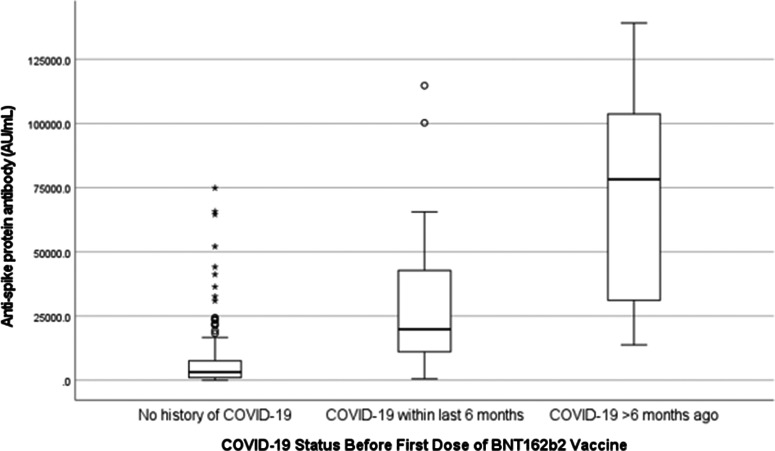
Comparison of antibody response to 2 doses of SARS-CoV-2 mRNA vaccine BNT162b2 based on previous COVID-19 status (N = 173).

In the remaining cohort of 173 patients who received 2 vaccine doses, the median age was 60 years (range, 28–88), 53.2% were men, and 85% were of Black race. The median time from the second vaccine dose to blood specimen collection was 35 days (range, 14–74) and the median anti-S level was 4,100 AU/mL (range, 0–139,121). Of the 173 patients, 7 (4%) did not have a serological response (anti-S, <50 AU/mL), 27 (15.6%) had a low response (anti-S, 50–839 AU/mL), and 139 (80.3%) had a sufficient antibody response (anti-S, ≥840 AU/mL) (Table 1). Also, 25 patients (15%) had a history of COVID-19 prior to SARS-CoV-2 vaccination; 24 of these (96%) had a sufficient antibody response and 1 (4%) had a low response. Antibody response was significantly greater in patients with prevaccination COVID-19 compared to patients without prior infection (median anti-S 31,195 AU/mL vs 3,165 AU/mL; *P* < .001). Antibody response was more robust if COVID-19 occurred >6 months prior to the first dose of vaccine (N = 9) compared to <6 months prior (N = 16) (median anti-S, 78,253 AU/mL vs 19,800 AU/mL; *P* < .001) (Fig. 1).

**Table 1.** Distribution of Serological Response Among Patients With Two Doses of SARS-CoV-2 mRNA Vaccine BNT162b2

Anti-spike Protein Antibody Level, AU/mL	All Patients (N = 173 No. (%)	Patients Without Prior COVID-19 (N = 147) No. (%)	Patients With Prior COVID-19 (N = 26) No. (%)
<50 (nonresponse)	7 (4)	7 (4.7)	0 (0)
50–839 (low response)	27 (15.6)	26 (17.7)	1 (3.8)
≥840 (sufficient response)	139 (80.3)	114 (77.6)	25 (96.2)

Moreover, 8 patients had a breakthrough COVID-19 infection 25–248 days after receiving the second vaccine dose, 7 of whom were Black, and all were on in-center HD (Table 2). Anti-S levels obtained 35–49 days after the second vaccine dose ranged from 404 AU/mL to 34,315 AU/mL. Only 1 patient had a low antibody response (< 840 AU/mL); she was hospitalized and died of severe COVID-19. Another patient on chronic prednisone (20 mg/day) for an autoimmune condition, and a previous history of COVID-19 (13 months pre-vaccination), developed COVID-19 8 months after the second vaccine dose and was hospitalized with moderate symptoms. The rest of the patients with breakthrough infection had mild symptoms of COVID-19 and did not require hospitalization.

**Table 2.** Characteristics of Patients Who Developed COVID-19 After Two Doses of SARS-CoV-2 mRNA Vaccine BNT162b2

Patient	Age, Years	Sex	Race	Time from Vaccine #2 to Anti-S Testing, Days	Time from Vaccine #2 to COVID-19, Days	Anti-S Level, AU/mL	Pre-vaccine COVID-19	Previous Organ Transplant	Immunosuppressive Medication	Previous Response to HBV Vaccination	Hospitalized Due to COVID-19	Death due to COVID-19
1	71	M	W	36	25	11135	No	No	No	Yes	No	No
2	86	F	B	49	32	34315	No	No	No	No	No	No
3	79	F	B	35	119	404	No	No	No	No	**Yes**	**Yes**
4	44	F	B	35	159	1696	No	No	No	Yes	No	No
5	45	M	B	35	193	15128	No	Yes	No	Yes	No	No
6	79	F	B	35	206	6136	No	No	No	Yes	No	No
7	57	M	B	33	247	6803	Yes^a^	No	Yes^b^	No	**Yes**	No
8	36	M	B	35	248	1538	No	Yes	No	Yes	No	No

a Previous COVID-19 was about a year prior to SARS-CoV-2 vaccination.

b On prednisone 20 mg daily for an autoimmune condition.

Note. M, male; F, female; B, black; W, white; anti-S, antibody against spike protein of SARS-CoV-2; HBV: hepatitis B virus.

In univariate analysis, factors significantly associated with insufficient vaccine response (anti-S < 840 AU/mL) included HIV infection with an absolute CD4 count < 200 cells/mcL (6% vs 0%), active malignancy (12% vs 3%), prior nonresponse to hepatitis B (HBV) vaccination (38% vs 18%), and darbepoietin dose > 60 µg/week (29% vs 10%) (Table 3). Presence of autoimmune disease (18% vs 7%), ESRD comorbidity index ≥ 5 (44% vs 28%), and receipt of any immunosuppressive medication (18% vs 7%) were marginally associated with insufficient vaccine response. Patients with hypertension requiring antihypertensive medications (91% vs 65%) and prevaccination COVID-19 (17% vs 3%) were significantly more likely to develop a sufficient antibody response to the vaccine. There was no difference in antibody response between patients on in-center HD and those on PD (median antibody level, 4,237 AU/mL and 3,242 AU/mL, respectively). Black race was not a risk factor for low vaccine response (79% vs 86%).

**Table 3.** Univariate Risk Factors for Insufficient Response to Two Doses of SARS-CoV-2 mRNA Vaccine BNT162b2 (N = 173)

Characteristics	All Patients (N = 173), No. (%)	Anti-S < 840 AU/mL (N = 34), No. (%)	Anti-S ≥840 AU/mL (N = 139), No. (%)	RR (95% CI)	*P* Value
Age ≥ 60 y	92 (53)	19 (56)	73 (53)	1.12 (0.61–2.05)	.73
Sex, male	92 (53)	19 (56)	73 (53)	1.12 (0.61–2.05)	.73
**Race**					
Black	147 (85)	27 (79)	120 (86)	Reference	
Other	26 (15)	7 (21)	19 (14)	1.47 (0.71–3.01)	.31
**Body mass index, kg/m^2^**					
Normal (18.5–24.9)	44 (25)	7 (21)	37 (27)	Reference	
Underweight (<18.5)	4 (2)	1 (3)	3 (2)	1.57 (0.25–9.78)	.63
Overweight (25–29.9)	49 (28)	10 (29)	39 (28)	1.28 (0.53–3.08)	.58
Obese (>30)	76 (44)	16 (47)	60 (43)	1.32 (0.59–2.97)	.50
**Etiology of ESRD**					
Diabetes mellitus	64 (37)	12 (35)	52 (37)	0.93 (0.49–1.75)	.82
Hypertension	116 (67)	20 (59)	96 (69)	0.70 (0.38–1.29)	.26
Glomerular diseases	14 (8)	2 (6)	12 (9)	0.71 (0.19–2.66)	1.00
Polycystic kidney disease	5 (3)	1 (3)	4 (3)	1.02 (0.17–6.03)	1.00
Other	49 (28)	12 (35)	37 (27)	1.38 (0.74–2.57)	.31
**Dialysis access in use**					
AVF	86 (49)	18 (53)	66 (48)	Reference	
AVG	40 (23)	6 (18)	34 (25)	0.70 (0.30–1.63)	.41
HD catheter	25 (15)	5 (15)	20 (14)	0.93 (0.39–2.26)	.88
PD catheter	24 (14)	5 (15)	19 (14)	0.97 (0.40–2.35)	.95
Dialysis Modality					
In-center HD	149 (86)	29 (85)	120 (86)	Undefined	
PD	24 (14)	5 (15)	19 (14)	1.07 (0.46–2.49)	1.00
Dialysis vintage					
<1 y	29 (17)	4 (12)	24 (17)	Reference	
1–3 y	63 (36)	15 (44)	48 (35)	1.73 (0.63–4.75)	0.29
>3 y	81 (47)	15 (44)	66 (48)	1.34 (0.49–3.72)	.57
**Comorbid conditions**					
Diabetes mellitus	84 (49)	14 (41)	70 (50)	0.74 (0.40–1.37)	.34
Hypertension requiring antihypertensive medications	148 (86)	22 (65)	126 (91)	0.31 (0.18–0.54)	<.001
Coronary artery disease	48 (28)	9 (27)	39 (28)	0.94 (0.47–1.86)	.85
Congestive heart failure	56 (32)	14 (41)	42 (30)	1.46 (0.80–2.68)	.22
Cerebrovascular accident	27 (16)	3 (9)	24 (17)	0.54 (0.18–1.64)	.30
Neurological disease	20 (12)	2 (6)	18 (13)	0.48 (0.12–1.85)	.37
Lung disease	26 (15)	6 (18)	20 (15)	1.21 (0.56–2.63)	.63
Cirrhosis	4 (2)	1 (3)	3 (2)	1.28 (0.23–7.18)	1.00
Active hepatitis C	6 (4)	2 (6)	4 (3)	1.74 (0.54–5.63)	.34
HIV with CD4 < 200 cells/mcL	2 (1)	2 (6)	0 (0)	Undefined	.04
Previous COVID-19 infection	25 (15)	1 (3)	24 (17)	0.18 (0.03–1.25)	.03
Sickle cell disease	3 (2)	0 (0)	3 (2)	Undefined	1.00
Active malignancy	8 (5)	4 (12)	4 (3)	2.75 (1.28–5.91)	.049
Autoimmune disease	16 (9)	6 (18)	10 (7)	2.10 (1.03–4.30)	.06
Prior renal transplantation	19 (11)	5 (15)	14 (10)	1.40 (0.62–3.18)	.54
Current nonrenal solid–organ transplantation	3 (2)	2 (6)	1 (1)	3.55 (1.50–8.33)	.10
Active on kidney transplant list	25 (15)	5 (15)	20 (14)	1.02 (0.44–2.39)	1.00
Lack of response to prior hepatitis B vaccination	38 (22)	13 (38)	25 (18)	2.20 (1.22–3.97)	.01
**ESRD comorbidity index**					.33
<5	119 (69)	19 (56)	100 (72)	Reference	
≥5	54 (31)	15 (44)	39 (28)	0.74 (0.96–3.16)	.07
**Medications**					
Systemic corticosteroids^a^	8 (5)	3 (9)	5 (4)	2.00 (0.77–5.16)	.19
Hydroxychloroquine	4 (2)	1 (3)	3 (2)	1.27 (0.23–7.14)	1.00
Biologic agent^b^	2 (1)	1 (3)	1 (1)	2.59 (0.63–10.71)	.36
Other immunosuppressant^c^	8 (5)	3 (9)	5 (4)	2.00 (0.77–5.16)	.19
Any immunosuppressive medication^d^	16 (9)	6 (18)	10 (7)	2.10 (1.03–4.30)	.06
Active chemotherapy	0	0	0		
ACEI or ARB	61 (35)	11 (32)	50 (36)	0.88 (0.46–1.68)	.69
Intravenous iron, any type or dose	122 (71)	25 (74)	97 (70)	1.16 (0.58–2.31)	.67
**Darbepoietin dose per week, µg**					
<60	149 (86)	24 (71)	125 (90)	Reference	
60–100	24 (14)	10 (29)	14 (10)	2.59 (1.42–4.71)	.003
**Laboratory data**					
Kt/V_urea_ ≥ 1.2 in HD, ≥ 1.7 in PD	…	34 (100)	138 (99)		1.00
Ferritin, ng/mL	…	897 (19–2,592)	872 (31–2,987)		.70
nPCR, g/kg/day	…	0.88 (0.49–1.47)	0.89 (0.30–1.79)		.72
**Hemoglobin, g/dL**					
≥12	21 (12)	4 (12)	17 (12)	Reference	
10–12	95 (55)	16 (47)	79 (57)	0.88 (0.33–2.38)	.81
9–10	34 (20)	6 (18)	28 (20)	0.93 (0.30–2.90)	.90
<9	23 (13)	8 (24)	15 (11)	1.83 (0.64–5.19)	.26
**Serum albumin, g/dL**					
>4	29 (17)	5 (15)	24 (17)	0.89 (0.37–2.17)	.81
3.5–4	109 (63)	21 (62)	88 (63)	Reference	
< 3.5	35 (20)	8 (24)	27 (19)	1.19 (0.58–2.44)	.64

Note. Anti-S, antibody against the spike protein of SARS-CoV-2; RR: relative risk; CI, confidence interval; ESRD, end-stage renal disease; AVF, arterio-venous fistula; AVG, arterio-venous graft; HD, hemodialysis; PD, peritoneal dialysis; HIV, human immunodeficiency virus; ACEI, angiotensin-converting enzyme inhibitor; ARB, angiotensin II receptor blocker; nPCR: normalized protein catabolic rate. Values are presented as no. (%) or median (range).

a All prednisone; dose = 5 mg (n = 6), 7.5 mg (n = 1), 20 mg (n = 1).

b Both adalimumab.

c Tacrolimus (5), azathioprine (2), leflunomide (1).

d Corticosteroids (prednisone), biologic agents (adalimumab), and other immunosuppressants (tacrolimus, azathioprine, leflunomide).

Variables significantly associated with vaccine non-response using the cutoff of 50 AU/mL are shown in Supplementary Table 1 and include prior nonresponse to HBV vaccination, chronic prednisone use, and darbepoietin dose >60 µg per week. Characteristics of nonresponders are shown in Supplementary Table 2.

Multivariate analysis of independent risk factors for insufficient vaccine response are shown in Table 4. The 25 patients with known prevaccination COVID-19 were excluded from the model since only 1 had a low response. Variables independently associated with insufficient vaccine response were ESRD comorbidity index score ≥5 and absence of prior HBV vaccination response. High-dose darbepoietin was marginally associated with insufficient vaccine response in the multivariable model.

**Table 4.** Independent Risk Factors for Insufficient Vaccine Response^a^

Factors	RR (95% CI)	*P* Value
ESRD comorbidity index ≥ 5	1.91 (1.09–3.34)	.024
Absence of prior HBV vaccination response	1.93 (1.12–3.34)	.018
Darbepoietin dose > 60 mcg per week	1.80 (0.99–3.27)	.06

Note. RR, relative risk; CI, confidence interval; ESRD, end-stage renal disease; HBV, hepatitis B virus.

a HIV infection with CD4 < 200 cells/µL was not included due to small cell sizes; active malignancy was included as a component of the ESRD comorbidity index; hypertension was excluded to focus on risk factors associated with insufficient vaccine response.

**Fig. 2.**
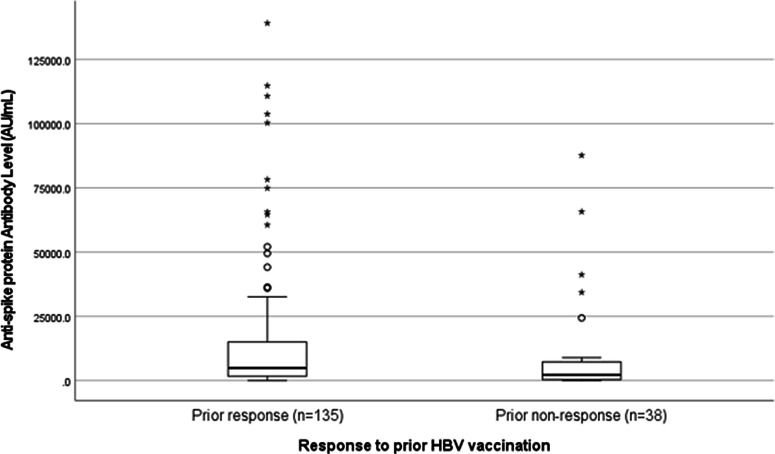
Comparison of antibody response to 2 doses of SARS-CoV-2 mRNA vaccine BNT162b2 based on previous hepatitis B vaccination response status (N = 173). Note. HBV, hepatitis B virus.

The relationships between absence of HBV vaccination response, hypertension, and COVID-19 vaccination response were investigated further. The median anti-S titer was significantly lower among patients without a prior HBV vaccination response compared to those with a prior response (2,173 AU/mL vs 4,905 AU/mL; *P* = .007) (Fig. 2). Patients with hypertension requiring antihypertensive medications were significantly less likely to be HBV vaccine nonresponders than patients without hypertension (19% vs 40%; *P* = .02) and had fewer years on dialysis (18% of patients with hypertension versus 8% without hypertension had been on dialysis for <1 year, and 43% of patients with hypertension versus 72% without hypertension had been on dialysis for >3 years [*P* = .03]).

## Discussion

In this single-center, US study of humoral response to 2 doses of SARS-CoV-2 mRNA vaccine BNT162b2, we observed a high seroconversion rate of 96% using the manufacturer recommended cutoff of anti-S >50 AU/mL in a cohort of predominantly Black dialysis patients. This finding is similar to that reported in studies in patients receiving maintenance dialysis from Europe and Israel.^10,21–23^ As seropositivity does not necessarily translate into protective immunity, we assessed humoral response using an anti-S level of 840 AU/mL as a cutoff for “sufficient response” because this value correlates with a neutralizing titer of 1:250, which corresponds to a 95% probability of viral neutralization and is likely predictive of protective immunity against COVID-19.^6,19^ We found that 20% of our study cohort fell below this threshold, suggesting that 2 doses of the vaccine may be ineffective in 1 of 5 patients in this dialysis population. Similar response rates were shown by 2 other US dialysis studies that utilized comparable neutralizing titers as the cutoff for protective immunity.^7,24^

Patients with prevaccination COVID-19 had heightened antibody response compared to those without prior COVID-19. This finding is consistent with published findings in both the general and dialysis population and supports the presence of immune memory and so called “hybrid immunity” to SARS-CoV-2.^19,25^ We also found a more vigorous antibody response in patients whose COVID-19 occurred >6 months prior to vaccination compared to those whose infection occurred <6 months before vaccination. This interesting phenomenon has been examined by Gaebler et al^26^ who have shown that memory B cells continue to evolve and display clonal turnover 6 months after COVID-19, producing antibodies with somatic hypermutation and more potent neutralizing activity.^26,27^ These B-cell clones expand markedly after vaccination, leading to a robust serological response. This observation may have implications in vaccine booster strategy in patients on dialysis.

In our cohort, 8 patients had a breakthrough COVID-19 infection 25–248 days after receiving the second vaccine dose (Table 2). The first 2 patients had their blood drawn for antibody titers 11–17 days after their COVID-19 diagnosis so the higher titers could be partly due to response to the infection itself. The third patient with breakthrough infection ∼4 months after the second vaccine dose did not attain sufficient antibody response, so infection was not surprising. For the rest, COVID-19 occurred >5 months after the second vaccine dose, implying that protective humoral immunity may have waned over time. This confirms a previous study in a maintenance HD population, which showed rapid antibody loss by 6 months after SARS-CoV-2 vaccination.^28^ In a recent study examining breakthrough infection in dialysis patients after SARS-CoV-2 vaccination, antibody titers decreased significantly 5–6 months after vaccination and breakthrough infection correlated with preinfection circulating antibody level,^29^ emphasizing the need for a vaccine booster around 6 months after the second vaccine dose in these patients. Of our 8 patients with breakthrough infection, 2 developed severe disease and the rest only had mild symptoms, suggesting that a sufficient antibody response after 2 doses of SARS-CoV-2 vaccine may be effective in preventing severe illness at 6–8 months.

In our cohort, factors associated with insufficient antibody response by univariate analyses included HIV infection with CD4 count < 200 cells/mcL, active malignancy, prior nonresponse to HBV vaccination, and use of high-dose erythropoiesis-stimulating agent (ESA). Multivariate analyses identified ESRD comorbidity index ≥5 and absence of prior HBV vaccination response as predictors of insufficient vaccine response. These findings suggest that sicker and more immunocompromised patients are less likely to respond to the vaccine. Prior nonresponse to HBV vaccination is a marker of immune dysfunction in ESRD patients. ESRD is an immunocompromising condition with impairment in both innate and adaptive immunity which can lead to attenuated response to all vaccines.^30–32^ High-dose ESA or ESA resistance is commonly associated with elevated inflammatory markers and has been negatively associated with response to HBV vaccination.^33^ However, the mechanisms by which ESA resistance leads to reduced vaccine responsiveness are yet to be elucidated. Also, patients with hypertension requiring blood pressure medications were more likely to develop a sufficient antibody response than those without hypertension. This has not been observed by others. In fact, hypertension has been shown to be a predictor of low vaccine response in nondialysis populations.^34,35^ The likely explanation for our finding is that the patients with hypertension in our study population could represent a healthier and less immunocompromised subgroup, and this is supported by our observation that patients with hypertension were less likely to be HBV vaccine nonresponders and had a shorter dialysis vintage.

Unlike other studies,^6,7,9,11,36,37^ older age, immunosuppressive therapy, longer dialysis vintage, and lower serum albumin were not independent predictors of humoral response in our cohort. One study showed that non-White race and Hispanic ethnicity were associated with lower response to mRNA vaccine.^7^ In our study, race was not associated with vaccine response.

Our study had several limitations. It was a single-center study with a small sample size and without a control group. Several studies have shown a blunted antibody response in patients on dialysis compared to nondialysis populations or healthy controls.^6,10,11,38^ We did not measure prevaccination antibody levels; therefore, we could not rule out the possibility of asymptomatic COVID-19 cases and subsequent misclassification in our analyses. We did not assess cellular immune response or evaluate the durability of humoral immunity. As our vaccination program was implemented several months ago, humoral response to the current and emerging variants was not assessed. Also, we only examined response to BNT162b2 vaccine and whether response to mRNA-1273 would be significantly different in our cohort is unknown. Lastly, because our patients are predominantly Black, results from this study may not be generalizable to non-Black patients.

In conclusion, most dialysis patients in our cohort seroconverted after 2 doses of BNT162b2 mRNA vaccine. However, 1 of 5 patients remained at risk for COVID-19 due to the inability to achieve sufficient protective immunity. Although SARS-CoV-2 vaccination is strongly recommended for all patients on maintenance dialysis, the relatively high rate of low vaccination response shown in this study emphasizes the need for a more effective vaccine strategy in this vulnerable population, perhaps with modification of vaccine dose and booster frequency, and with mitigating strategies such as ring vaccination. Our results also highlight the need to identify the subgroup of dialysis patients at risk for inadequate vaccine response via serological testing and the importance of maintaining preventative measures such as social distancing and mask wearing until a protective immune response is attained.

## Supplementary material

For supplementary material accompanying this paper visit https://doi.org/10.1017/ash.2022.31. click here to view supplementary material
