# Supplementary material for: Predictors of humoral response to SARS-CoV-2 mRNA vaccine BNT162b2 in patients receiving maintenance dialysis
Source: Antimicrob Steward Healthc Epidemiol. 2022 Mar 23;2(1):e48. doi: 10.1017/ash.2022.31 (PMC9615013; doi:10.1017/ash.2022.31)
Supplement: Supplementary file 1 [file S2732494X22000316sup001.pdf]

# Supplemental Material

## Table of Contents

1. **Supplemental Table 1**

2. **Supplemental Table 2**

**Supplemental Table 1. Univariate risk factors for vaccine non-response two doses of SARS-CoV-2 mRNA vaccine BNT162b2 (N=173)**

| Characteristics | Anti-S < 50 AU/mL N=7 | Anti-S ≥ 50 AU/mL N=166 | RR (95% CI) | p |
|---|---|---|---|---|
| Age ≥ 60 years | 3 (43) | 89 (54) | 0.66 (0.15 – 2.86) | 0.71 |
| Men | 5 (71) | 87 (52) | 2.20 (0.44 – 11.04) | 0.45 |
| Race | | | | |
| Black | 5 (71) | 142 (86) | Reference | |
| Other | 2 (29) | 24 (15) | 2.26 (0.46 – 11.04) | 0.28 |
| Body Mass Index ($kg/m^2$) | | | | |
| Normal or underweight (<25) | 4 (40) | 48 (28) | Reference | |
| Overweight or obese (≥ 25) | 6 (60) | 123 (72) | 2.30 (0.28 – 18.64) | 0.68 |
| Etiology of ESRD | | | | |
| Diabetes mellitus | 0 (0) | 64 (39) | Undefined | 0.047 |
| Hypertension | 4 (57) | 112 (68) | 0.66 (0.15 – 2.83) | 0.69 |
| Glomerular diseases | 0 (0) | 14 (8) | Undefined | 1.00 |
| Polycystic kidney disease | 0 (0) | 5 (3) | Undefined | 1.00 |
| Other | 4 (57) | 45 (27) | 3.37 (0.78 – 14.53) | 0.10 |
| Dialysis Access in Use | | | | |
| AVF | 3 (43) | 81 (49) | Reference | |
| AVG | 1 (14) | 39 (24) | 0.67 (0.07-6.18) | 0.72 |
| HD catheter | 2 (29) | 23 (14) | 2.82 (0.51-15.60) | 0.23 |
| PD catheter | 1 (14) | 23 (14) | 1.04 (0.11-9.55) | 0.97 |
| Dialysis Modality | | | | |
| In-center HD | 6 (86) | 143 (86) | Reference | |
| PD | 1 (14) | 23 (14) | 1.03 (0.13 – 8.22) | 1.00 |
| Dialysis Vintage, years | | | | |
| <1 | 3 (43) | 25 (15) | Reference | |
| 1-3 | 2 (29) | 61 (37) | 0.31 (0.05 – 1.84) | 0.20 |
| >3 | 2 (29) | 79 (48) | 0.24 (0.04 – 1.43) | 0.12 |
| Comorbid conditions | | | | |
| Diabetes | 0 (0) | 84 (51) | Undefined | 0.01 |
| Hypertension requiring anti-hypertensive medications | 2 (29) | 146 (88) | 0.07 (0.01 – 0.33) | <0.001 |
| Coronary artery disease | 2 (29) | 46 (28) | 1.04 (0.21 – 5.19) | 1.00 |
| Cerebrovascular accident | 1 (14) | 26 (16) | 0.90 (0.11 – 7.14) | 1.00 |
| Congestive heart failure | 4 (57) | 52 (31) | 2.79 (0.65 – 12.03) | 0.22 |
| Neurological disease | 1 (14) | 19 (11) | 1.28 (0.16 – 10.05) | 0.58 |
| Lung disease | 0 (0) | 26 (16) | Undefined | 0.60 |
| Cirrhosis | 0 (0) | 4 (2) | Undefined | 1.00 |
| Active hepatitis C | 0 (0) | 6 (4) | Undefined | 1.00 |
| HIV with CD4 < 200 cells/mcL | 1 (14) | 1 (1) | 14.25 (2.90 – 70.11) | 0.08 |
| Previous COVID-19 infection | 0 (0) | 25 (15) | Undefined | 0.60 |
| Sickle cell disease | 0 (0) | 3 (2) | Undefined | 1.00 |
| Active malignancy | 1 (10) | 7 (4) | 3.44 (0.47 – 25.26) | 0.29 |
| Autoimmune disease | 1 (14) | 15 (9) | 1.64 (0.21 – 12.75) | 0.50 |
| Prior renal transplantation | 2 (29) | 17 (10) | 3.24 (0.68 – 15.57) | 0.17 |
| Current non-renal solid organ transplantation | 1 (14) | 2 (1) | 9.44 (1.59 – 56.17) | 0.12 |
| Active on kidney transplant list | 1 (14) | 24 (15) | 0.99 (0.12 – 7.85) | 1.00 |
| Lack of response to prior hepatitis B vaccination | 4 (57) | 34 (21) | 4.74 (1.11 – 20.26) | 0.04 |
| ESRD comorbidity Index | | | | |

| | | | | |
|---|---|---|---|---|
| <5 | 3 (43) | 116 (70) | Reference | |
| ≥5 | 4 (57) | 50 (30) | 2.94 (0.68 – 12.68) | 0.21 |
| Medications | | | | |
| Systemic corticosteroids[a] | 2 (29) | 6 (4) | 8.25 (1.88 – 36.18) | 0.04 |
| Hydroxychloroquine | 0 (0) | 4 (2) | Undefined | 1.00 |
| Biologic agent[b] | 0 (0) | 2 (1) | Undefined | 1.00 |
| Other immunosuppressant[c] | 1 (14) | 7 (4) | 3.44 (0.47 – 25.26) | 0.29 |
| Any immunosuppressive medication[d] | 2 (29) | 14 (8) | 3.93 (0.83 – 18.62) | 0.13 |
| Active chemotherapy | 0 | 0 | | |
| ACEI or ARB | 1 (14) | 60 (36) | 0.31 (0.04 – 2.48) | 0.42 |
| Intravenous iron, any type or dose | 5 (71) | 117 (71) | 1.05 (0.21 – 5.21) | 1.00 |
| Darbepoietin dose per week (mcg) | | | | |
| < 60 | 2 (29) | 147 (89) | Reference | |
| 60-100 | 5 (71) | 19 (11) | 15.52 (3.19 – 75.51) | < 0.001 |
| Laboratory Data | | | | |
| $Kt/V_{urea} \geq 1.2$ in HD, ≥ 1.7 in PD | 7 (100) | 165 (99) | | 1.00 |
| Ferritin (ng/mL) | 1097 (138-2592) | 876 (19-2987) | | 0.30 |
| nPCR (g/kg/day) | 1.20 (0.57-1.41) | 0.88 (0.30-1.79) | | 0.20 |

Values are presented as n (%) or median (range).

Anti-S: antibody against the spike protein of SARS-CoV-2; RR: relative risk; CI: confidence interval; ESRD: end-stage renal disease; AVF: arterio-venous fistula; AVG: arterio-venous graft; HD: hemodialysis; PD: peritoneal dialysis; HIV: human immunodeficiency virus; COVID-19: Coronavirus disease 2019; ACEI: angiotensin-converting enzyme inhibitor; ARB: angiotensin II receptor blocker; nPCR: normalized protein catabolic rate

[a] All prednisone; dose=5 mg (n=6), 7.5 mg (n=1), 20 mg (n=1)
[b] Both adalimumab
[c] Tacrolimus (5), azathioprine (2), leflunomide (1)
[d] Corticosteroids (prednisone), biologic agents (adalimumab), and other immunosuppressants (tacrolimus, azathioprine, leflunomide)

**Supplemental Table 2. Characteristics of Non-responders (those who did not seroconvert) After Receiving Two Doses of SARS-CoV-2 mRNA Vaccine BNT162b2**

| Patient | Age (years) | Sex | BMI | Anti-S level (AU/mL) | Previous COVID-19 | Non-renal transplant | Renal Transplant | Immunosuppressive Medication | Dialysis Modality |
|---|---|---|---|---|---|---|---|---|---|
| 1 | 61 | M | 38.6 | 0 | No | No | No | No | In-center HD |
| 2 | 41 | M | 21.6 | 22.2 | No | No | Yes | No | In-center HD |
| 3 | 43 | F | 55.5 | 39.3 | No | No | No | No | In-center HD |
| 4 | 70 | M | 39.9 | 0 | No | Yes, heart | No | Yes | In-center HD |
| 5 | 58 | F | 40.7 | 5.9 | No | No | No | No | In-center HD |
| 6 | 55 | M | 34.1 | 6.6 | No | No | No | No | In-center HD |
| 7 | 73 | M | 29.5 | 3.7 | No | No | Yes | Yes | PD |

Anti-S: Antibody against spike protein of SARS-CoV-2; M: Male; F: Female; BMI: Body mass index; COVID-19: Coronavirus 2019; HD: Hemodialysis; PD: Peritoneal dialysis
